# Gold nanochips with molecularly imprinted polymer coating for sensing explosives: a surface-enhanced Raman scattering approach

**DOI:** 10.1098/rsos.251279

**Published:** 2025-09-24

**Authors:** Petro Demydov, Andrii Lopatynskyi, Nazar Mazur, Oksana Isaieva, Vitalii Lytvyn, Mariia Khutko, Volodymyr Yukhymchuk, Gennady Monastyrsky, Volodymyr Chegel

**Affiliations:** ^1^ V. Lashkaryov Institute of Semiconductor Physics NAS of Ukraine, Kyiv, Ukraine; ^2^ Educational and Research Institute of Physics and Technology, National Technical University of Ukraine Igor Sikorsky Kyiv Polytechnic Institute, Kyiv, Ukraine

**Keywords:** surface-enhanced Raman scattering, localized surface plasmon resonance, gold nanostructures, nanochip, molecularly imprinted polymer, sensor, explosives

## Abstract

This research is aimed at developing a sensor that is both sensitive and selective for detecting explosives, and to examine its surface-enhanced Raman scattering (SERS) response. Molecularly imprinted polymers (MIPs), commonly used as synthetic receptors in sensors for the selective detection of molecules without prior analytic treatments, have been combined with optical sensors. This integration, especially in combination with localized surface plasmon resonance (LSPR) technology, represents a promising strategy for identifying explosives. Specifically, we considered a sensor based on a gold nanostructure array deposited onto a glass substrate (gold nanochip), followed by the fabrication of an MIP layer using the photochemical polymerization method with 4-nitrophenol (4-NP) as a template molecule. The selectivity of the sensor was assessed by comparing the SERS and LSPR responses against 4-NP and other chemical analogues of nitro-containing explosives. Notably, this technique not only ensures sensor selectivity but is also capable of detecting analytes at concentrations as low as 100 μM.

## 1. Introduction

In recent years, identifying explosives has become increasingly important for national security. This process is critical for effective mine clearance, locating unexploded munitions, enforcing border and customs regulations, and monitoring environmental contamination. As a result, there has been a significant rise in research aimed at both innovating new methods and refining existing techniques to develop more sensitive, affordable and straightforward devices for detecting explosive residues [1,2].

Military explosives generally contain aliphatic and aromatic molecules with nitro functional groups (NO_2_). The strong oxidizing properties of NO_2_ groups trigger fast exothermic reactions that produce intense heat and gas expansion, creating explosive force and shockwaves [3]. Current methods for detecting explosive molecules that contain nitro groups are diverse and sophisticated, utilizing both chemical and physical techniques [4]. For example, colorimetric sensors change colour in the presence of nitroaromatic compounds, providing a visual indication of explosives [5]. Another prominent method involves the use of ion mobility spectrometry, which can quickly and effectively detect traces of explosives in air by measuring the mobility of gas-phase ions [6]. Most techniques face limitations concerning cost, specificity, portability and operational requirements, which must be carefully managed depending on the application. Therefore, the research for simple, precise, portable and selective technology for explosive detection is ongoing.

Molecularly imprinted polymers (MIPs) are specialized porous materials designed with unique recognition abilities for specific template molecules [7]. These materials possess selective binding sites that are essentially cavities or pores shaped by the removal of the template molecule following the polymerization process. These precisely formed sites are capable of identifying and binding target molecules that share the same size, shape, structure and properties as the original template. As a result, MIPs are exceptionally effective in selectively capturing and analysing chemical compounds [8]. This specificity makes them highly valuable in various applications, including sensing, separation and biomedicine, where precise molecular recognition is crucial [9,10]. Their ability to selectively bind target molecules underpins their widespread use in fields like environmental monitoring, pharmaceuticals and food safety.

The phenomenon of localized surface plasmon resonance (LSPR) occurs on a surface of metal with irregularities or nanoparticles with sizes that are smaller than the wavelength of the incident radiation. In this case, the movement of electrons in the metal results in the formation of a dipole that oscillates with the frequency of the exciting electric field. When the oscillation frequency of the incident light matches the oscillation frequency of the localized surface plasmons of the particle, resonance absorption and scattering of light are observed, resulting in an enhancement of local electromagnetic field strength. The intensity, position and width of the LSPR peak depend on the material of the nanoparticles, shape, size, the distance between them and the refractive index of the surrounding medium [11–14]. It has been found that the best field enhancement is achieved with large, densely packed nanostructures, while continuous, smooth films provide the weakest field enhancement [15]. Studies have demonstrated the dependence of field enhancement on the distance between the nanostructures themselves due to the plasmon coupling effect. For example, when a fluorophore molecule is placed between two adjacent nanoparticles, the fluorescence enhancement of such a system is several times greater than when the molecule is placed near a single nanostructure due to the increased electromagnetic field intensity in the two-nanoparticle system [16]. Because of that fact, arrays of nanostructures supporting LSPR are the most suitable for plasmonic enhancement techniques.

Surface-enhanced Raman scattering (SERS) is an advanced analytical method that provides the ability to detect chemical entities quantitatively with extreme molecular precision and the sensitivity to identify single molecules [17] due to the electromagnetic field enhancement provided by LSPR excitation. While this technique offers significant benefits, its practical use has historically been restricted by its expensive nature. Nonetheless, recent advancements in creating multifunctional SERS substrates have opened new possibilities [18]. These innovative substrates, which include flexible designs, combine separation and enhancement, integrate calibration and enhancement and feature enhancement with regeneration capabilities, have demonstrated considerable potential, specifically for explosives detection [19,20] and point-of-care diagnostics [21]. These developments could greatly expand the applicability of SERS in various scientific and industrial fields, potentially making this powerful technique more accessible and cost-effective for broader use.

Previous research [22] has shown that combining MIP and SERS can be used to detect 0.4 mM of 2,4,6-trinitrotoluene (TNT) with high selectivity; compared to 2,4-dinitrotoluene and 2-nitrotoluene, a more than 10 times larger SERS response was shown to TNT, which was the template during MIP synthesis. For this, a glass coated with spherical silver particles was used as a SERS substrate, and xerogel was used as an MIP base [22]. Later, the same authors have shown that the 3σ limit of detection can be as low as 3 μM for TNT when using commercially available Klarite SERS substrate [23]. Molecular imprinting-based SERS detection strategies have also been proposed for other types of analytes, ranging from large-sized proteins [24] to small molecules like insecticides [25], which emphasizes the broad scope of potential applications for the combined MIP-SERS technique. For example, reproducible quantitation of the cyanobacteria-specific protein phycocyanin at the level down to 2.6 × 10^–3^ μg l^–1^ was reported, and practical applicability for analysing crude waterway samples was confirmed for polydopamine-based MIP with gold nanostars as a SERS active substrate [24]. In another study, MIP was combined with electrochemical SERS to achieve specific recognition and a limit of detection down to 3.2 nM for insecticide acetamiprid [25].

It was also shown that MIP can be combined with the LSPR technique for sensing explosives by monitoring light absorbance in different noble nanoparticle-based systems. For example, silver nanoparticles embedded in an MIP that was imprinted with 3-nitrotoluene (3-NT) as a template have been used to detect 54.8 ng of 3-NT with a sensitivity of 24.0% ± 3.0% [26]. Gold nanochips containing spheroid gold nanoparticles coated with acrylamide-based MIP exhibited detection limits of 1 pM in aqueous solution and 0.1 ppm in gaseous state against 4-nitrophenol (4-NP) [27]. For such nanochips, the spectral position of LSPR and accompanying electromagnetic field enhancement can be manipulated by varying the thickness of the deposited noble metal thin film and annealing time to transform the film into nanoparticles [28]. Although the LSPR technique shows less selectivity towards detecting explosives when compared to SERS, it potentially demonstrates higher sensitivity.

In this article, we present gold nanochips with MIP coating for explosives sensing used in a measurement technique that combines the strengths of SERS and LSPR analyses, which allows us to achieve sensitive and selective detection of 4-NP, a chemical analogue of TNT explosive. We believe this study will contribute to the development of an alternative method of detecting explosives, which could be useful for monitoring the contamination of soil and water resources and for facilitating humanitarian demining.

## 2. Experimental

### 2.1. Materials

All chemicals were purchased from Sigma-Aldrich and used as received, except for UV-sensitive polymerization initiator that was custom synthesized [29]. Ultrapure deionized water (type I, *R* = 18.2 MΩ cm) from the water purification system Adrona B30 Bio was used for the preparation of the aqueous solutions. 2-Propanol was used to dissolve water-insoluble chemicals before preparing aqueous solutions to the desired concentration.

### 2.2. Synthesis

The current method involves creating plasmonic nanochips by forming random arrays of gold nanostructures (AuNS) on glass slides [27,28]. These nanochips offer advantages over colloidal gold nanoparticles, notably in their resistance to aggregation, owing to their attachment to a solid substrate. The fabrication process started with glass slides measuring 13 mm × 25 mm × 1 mm, used as substrates. Initially, these substrates were cleansed in an ultrasonic bath with surfactants, followed by a treatment with ‘piranha solution’ (a mixture of H_2_SO_4_ and H_2_O_2_) for 30 min. Subsequently, the slides were thoroughly rinsed three times with deionized water and dried with a flow of nitrogen gas.

**Figure 1.**
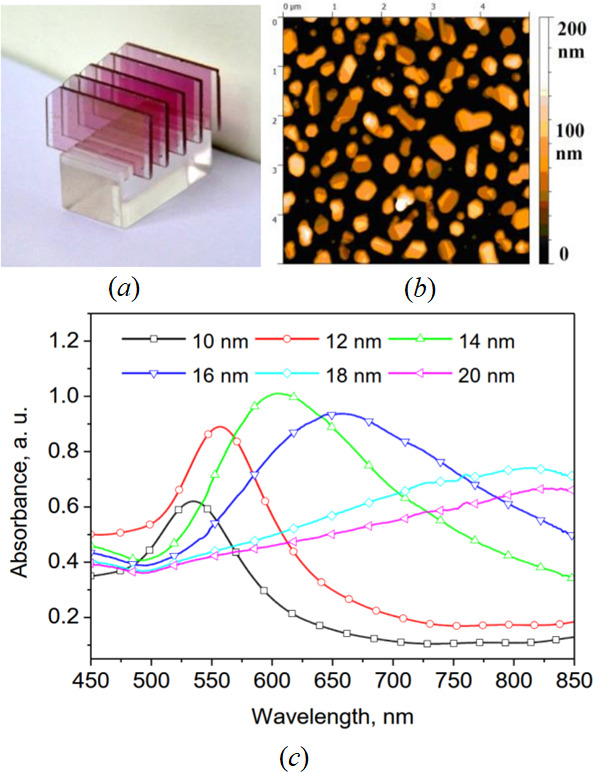
(*a*) Photograph of Au nanochips. (*b*) Microscopic image of AuNS on the surface of nanochip. (*c*) Light extinction spectra of Au nanochips fabricated from gold island films of different mass thickness. Reprinted from [27] under a Creative Commons Attribution-NoDerivatives 4.0 International License.

Gold island films were then deposited onto substrates using a thermal vacuum evaporation technique under a pressure of 10^−3^ Pa, with a deposition rate between 0.11 and 0.14 nm s^−1^. The chosen mass thickness for the gold island film was between 10 and 12 nm, facilitating the formation of distinct, well-defined AuNS that exhibit a pronounced LSPR spectral band after thermal annealing at 550°C for 2 h in an air atmosphere (figure 1) [27].

**Figure 2.**
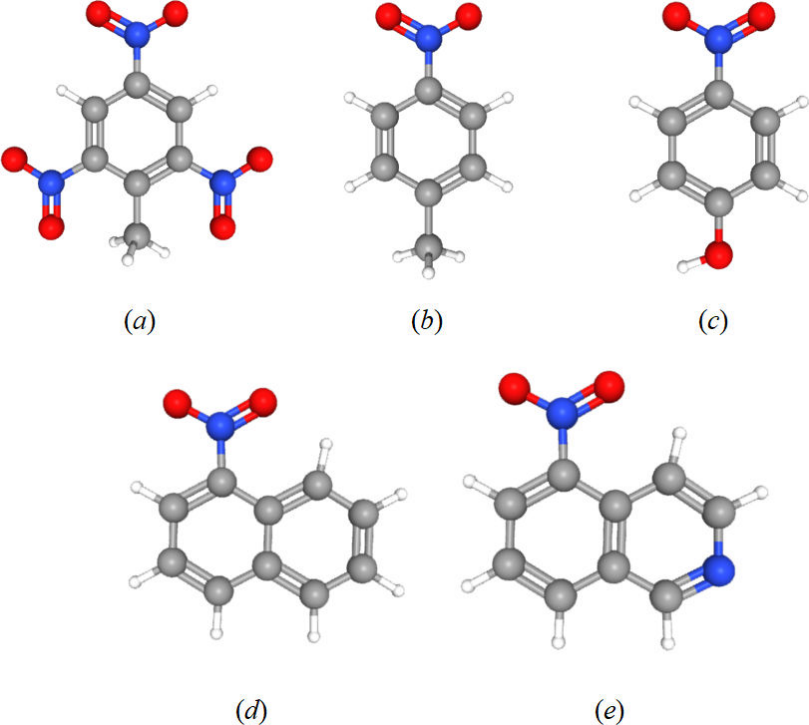
The molecular structure of (*a*) TNT, (*b*) 4-NT, (*c*) 4-NP, (*d*) 1-NN and (*e*) 5-NI. Color codes used for distinguishing atoms of chemical elements: oxygen – red, nitrogen – blue, carbon – grey, hydrogen – white. Data deposited in or computed by PubChem [32].

For the synthesis of MIP-coated nanochips, the surface of AuNS was initially functionalized with a layer of 3-mercaptopropyl diethylcarbamodithioate, which served as a UV-sensitive initiator for the polymerization process [27,29], by immersing the nanochip into a saturated 2-propanol solution of initiator overnight with subsequent drying under nitrogen flow. This was followed by UV-induced photochemical polymerization of a nitrogen-purged mixture of water-compatible acrylamide monomers selected from monomer libraries (acrylamide, *N*,*N′*-methylenebis(acrylamide) and *N*-(3-aminopropyl)methacrylamide hydrochloride) [30,31] in the presence of 4-NP as a template for 1 h. The choice of 4-NP as a chemical analogue of explosive due to its structural similarity to the explosive TNT (figure 2) was strategic, enhancing the nanochips' ability to recognize nitro-group containing explosives. 4-nitrotoluene (4-NT), 1-nitronaphthalene (1-NN) and 5-nitroisoquinoline (5-NI) as chemical analogues of explosive molecules were also used to test the selectivity of 4-NP-imprinted MIP substrate at a concentration of 100 μM.

**Figure 3.**
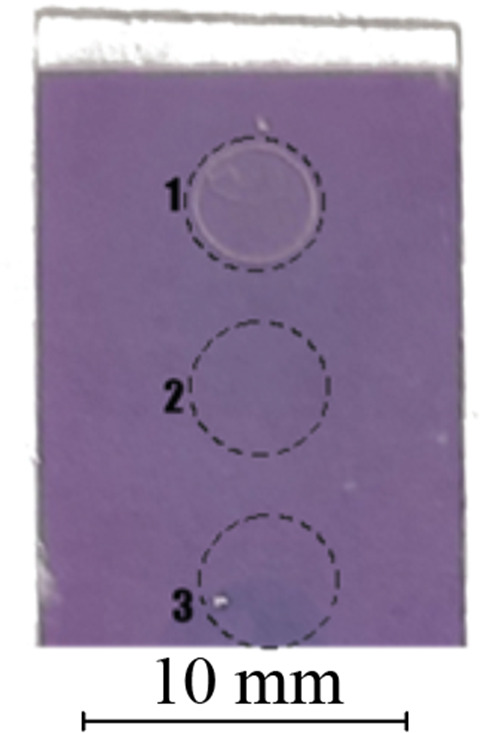
Photograph of Au nanochip with MIP coating and three indicated measurement spots: (1) 4-NP, (2) only MIP and (3) 4-NT.

Finally, the polymer-coated Au nanochip was washed in a solution of 4-(2-hydroxyethyl)piperazine−1-ethanesulfonic acid to remove any remaining explosive analogue template. After drying, the prepared MIP-coated nanochip was ready for use in explosives detection, showcasing its potential for sensitive and specific sensing applications. The image of the fabricated nanochip is shown in figure 3.

### 2.3. Characterization

Raman spectra were excited with a 457 nm solid-state laser (laser power is 12 mW, laser beam size is 100 μm) and acquired using a single-stage spectrometer MDR-23 (LOMO) equipped with a cooled CCD detector (Andor iDus 401A, UK). The laser power density on the samples was less than 10^3^ W cm^−2^ to preclude any thermal or photo-induced modification of the samples. A spectral resolution of 4 cm^−1^ was determined from the Si phonon peak width of a single-crystal Si substrate. The Si phonon peak position of 520.5 cm^−1^ was used as a reference for determining the position of the peaks in the Raman/SERS spectra of the analyte.

Light extinction measurements were carried out using a compact LSPR spectrometer ‘NanoPLASMON-003’ (ISP NASU, Ukraine) in a spectral range from 400 to 800 nm. This spectrometer is compatible with nanochips of various sizes (within the limits of a 1 inch × 3 inch standard plain microscopic slide) and allows measurements at various points of nanochip’s sensitive surface area. Unpolarized light from a tungsten–halogen light source was incident normally on the nanochip surface and collected using an optical fibre connected to a built-in miniature CCD spectrometer. Absorbance spectra were measured with an integration time of 10 ms and averaged over 100 consecutive spectral readouts.

## 3. Results and discussion

### 3.1. SERS study

SERS spectra of the plasmonic sensors based on Au nanochips with MIP coating (AuNS–MIP) were obtained in the presence of solutions of 4-NP, 4-NT, 1-NN and 5-NI molecules on their surface. Lasers with wavelengths λ_exc_ of 457 and 532 nm were considered for the analysis. However, the laser with a wavelength of 532 nm showed SERS response with a much lower signal-to-noise ratio, so further research was conducted with λ_exc_ = 457 nm.

**Figure 4.**
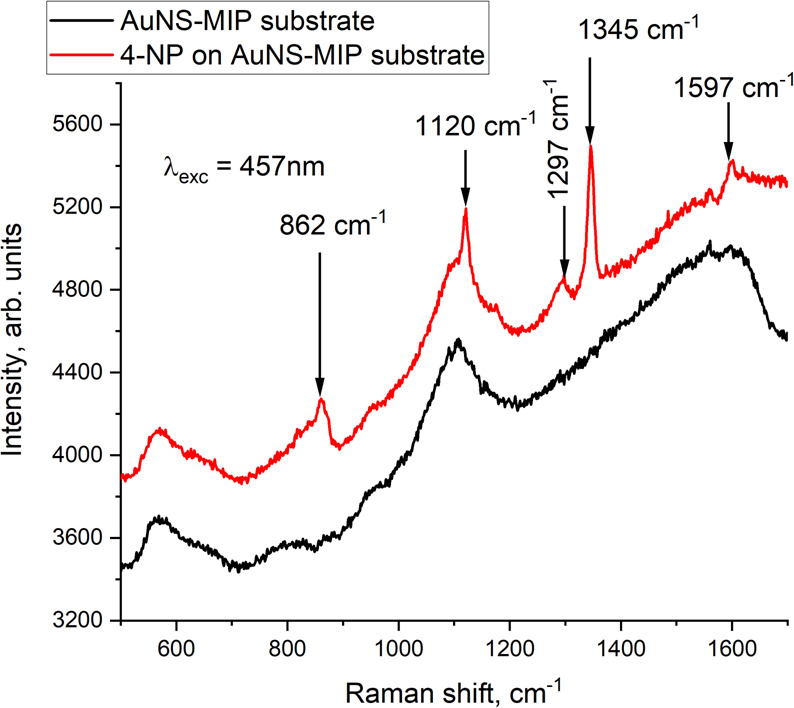
SERS spectra before (black) and after (red) addition of 100 µM 4-NP solution onto the surface of the 4-NP-imprinted AuNS–MIP plasmonic sensor.

Figure 4 shows the SERS response of a 4-NP-imprinted AuNS–MIP plasmonic sensor to the deposition of 100 µM 4-NP solution onto its surface. As can be seen, new peaks appear at positions 862, 1120, 1297, 1345 and 1597 cm^−1^ of the spectrum. Comparison with literature shows that the peak at 862 cm^−1^ corresponds to the bending deformation of the NO_2_ group and the stretching of C–C bonds. The peak at 1120 cm^−1^ is associated with C–H bending; the one at 1297 cm^−1^ is associated with C–O stretching and C–H bending deformations; and the one at 1345 cm^−1^ is associated with symmetrical vibrations of the NO_2_ group. The peak at 1597 cm^−1^ corresponds to the stretching of the benzene ring [33,34]. Thus, the molecular fingerprint of the 4-NP molecule was successfully identified using a 4-NP-imprinted AuNS–MIP plasmonic sensor.

**Figure 5.**
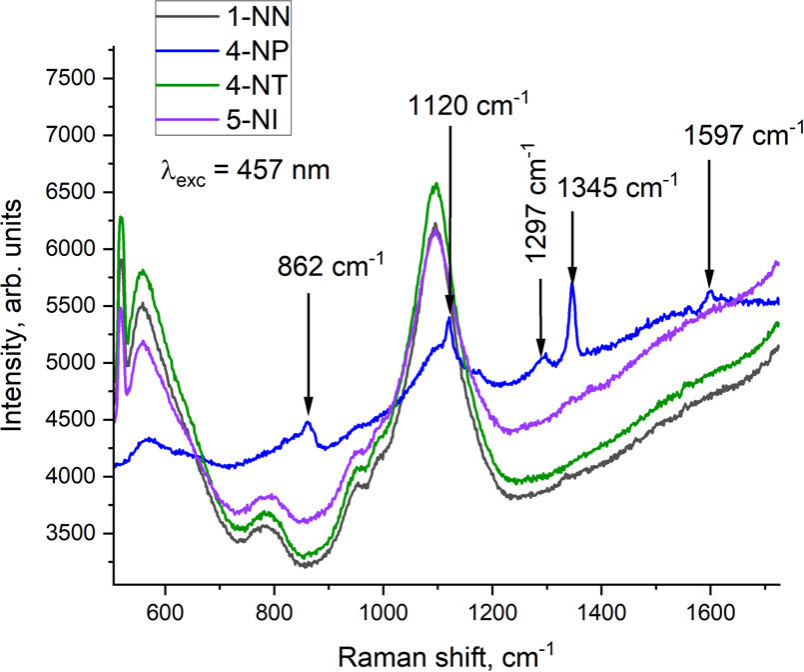
SERS spectra of different analytes on the surface of the 4-NP-imprinted AuNS–MIP plasmonic sensor.

Figure 5 shows the SERS response of a 4-NP-imprinted AuNS–MIP plasmonic sensor to the deposition of different analytes (4-NP, 4-NT, 1-NN and 5-NI) onto its surface. As can be seen, no new peaks emerge after other analytes addition as compared to the 4-NP analyte, which has typical peaks at 862, 1120 and 1345 cm^−1^. This corresponds to the absence of new chemical bond formation between analytes other than 4-NP and the MIP surface, thus confirming the selective response of 4-NP-imprinted MIP substrate to 4-NP only. It should be noted that the AuNS–MIP plasmonic sensor allows distinguishing 4-NP and 4-NT molecules that have a similar structure and differ only in one functional group, which, to the best of our knowledge, was previously possible only in more complicated sensor architectures based on fluorescence or electrochemical detection techniques [35,36].

**Figure 6.**
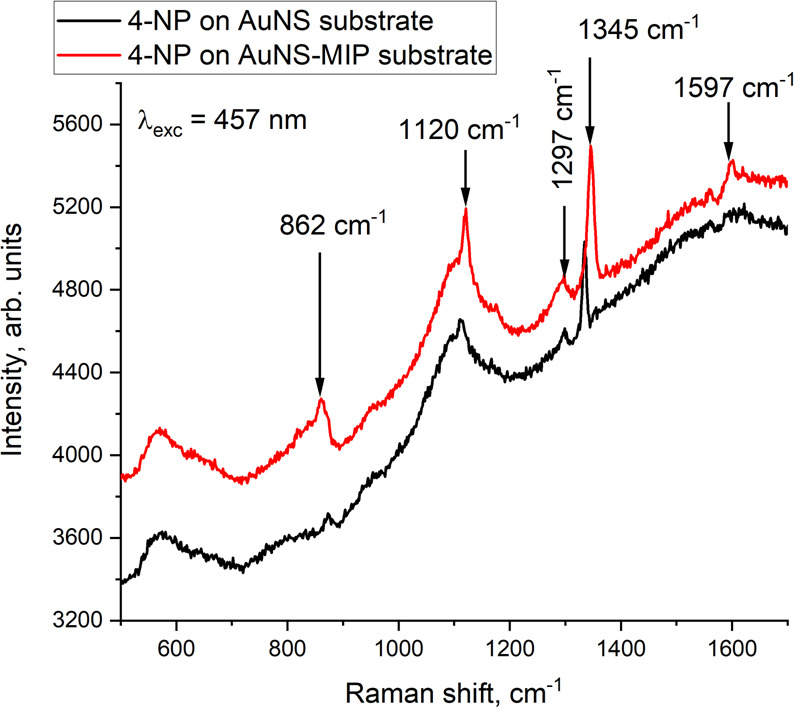
SERS spectra of 4-NP on the surface of Au nanochip without MIP coating (black) and Au nanochip with 4-NP-imprinted MIP coating (red).

SERS response of the Au nanochip without MIP coating and the Au nanochip with 4-NP-imprinted MIP coating to the presence of 4-NP was also compared. As shown in figure 6, the Au nanochip with MIP coating exhibits a more intense response, and the corresponding peaks are higher than those for the Au nanochip without MIP coating, which implies the role of MIP in enhancing the SERS response due to facilitation of spatial arrangement of analyte molecules in imprinted binding sites [37] around AuNS comprising the nanochip.

### 3.2. LSPR study

**Figure 7.**
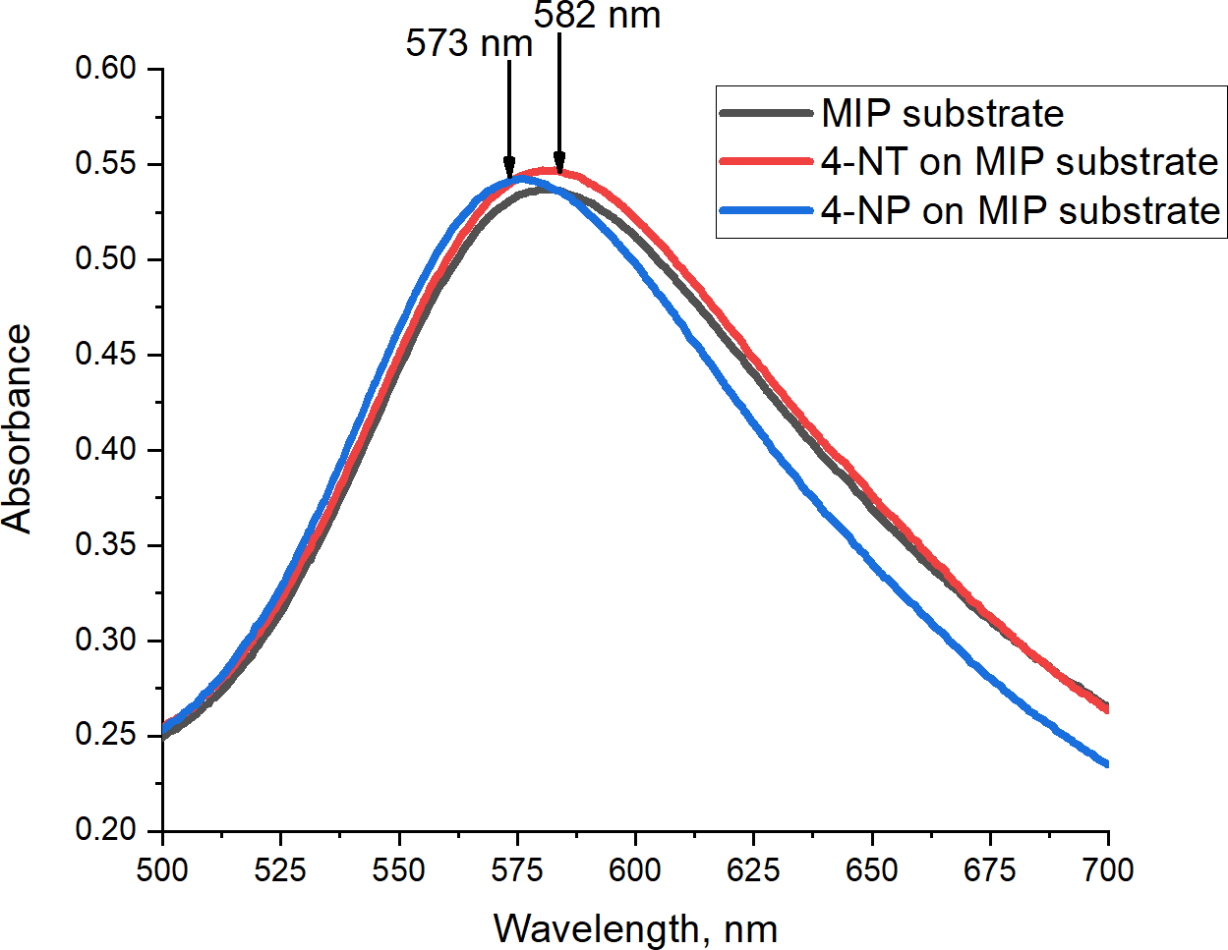
Light extinction spectra of AuNS–MIP plasmonic sensor for free 4-NP-imprinted MIP (black) and MIP with deposited 4-NP (blue) and 4-NT (red). Arrows indicate the LSPR peak positions.

Using the same AuNS–MIP plasmonic sensor, light extinction measurements were also performed at the measurement spots containing free 4-NP-imprinted MIP and MIP with deposited 4-NP and 4-NT, and LSPR bands in light extinction spectra were characterized. As is shown in figure 7, LSPR peaks for free MIP and MIP with deposited 4-NT exhibit similar wavelength positions of 582 nm, while the LSPR peak for MIP with deposited 4-NP positioned at 573 nm demonstrates a noticeable 9 nm blueshift, indicating a modification of the LSPR response due to the presence of 4-NP molecules bound to MIP. These variations in the LSPR response indicate the significant MIP interaction with 4-NP molecules and can be explained as MIP swelling at binding [38], affecting the plasmon resonance properties of AuNS and again emphasizing the selective response of 4-NP-imprinted MIP substrate to 4-NP molecules only.

## 4. Conclusions

We have shown that the AuNS–MIP sensor shows both highly selective and sensitive SERS responses at an excitation wavelength of 457 nm against explosive analogue molecules. Specifically, the sensor allows distinguishing 4-NP and 4-NT molecules that have a similar structure and differ only in one functional group. The AuNS–MIP sensor was demonstrated to exhibit a stronger SERS response in comparison to an AuNS sensor without MIP coating. The AuNS–MIP sensor readily detects 4-NP at a concentration of 100 µM, and it can detect even lower concentrations. Similar selectivity results were shown for LSPR response, indicating the universal nature of selective MIP interaction with only the molecules closely resembling the template used for MIP fabrication.

## Data Availability

The data supporting the findings of this study are available through Dryad [39].
